# Treatment with Direct-Acting Antivirals in Patients with HCV Infection After Liver Transplantation

**DOI:** 10.3390/jcm15010346

**Published:** 2026-01-02

**Authors:** Michał Brzdęk, Dorota Zarębska-Michaluk, Olga Tronina, Łukasz Laurans, Ewa Janczewska, Dorota Dybowska, Anna Parfieniuk-Kowerda, Magdalena Tudrujek-Zdunek, Jolanta Białkowska-Warzecha, Justyna Janocha-Litwin, Robert Flisiak

**Affiliations:** 1 Collegium Medicum, Jan Kochanowski University, 25-317 Kielce, Poland; 2 Department of Gastroenterology, Medical University of Lodz, 92-213 Lodz, Poland; 3 Department of Infectious Diseases and Allergology, Collegium Medicum, Jan Kochanowski University, 25-317 Kielce, Poland; dorota1010@tlen.pl; 4 Lazarski University, 02-662 Warsaw, Poland; olgatronina@wp.pl; 5 Department of Infectious Diseases, Hepatology and Liver Transplantation, Pomeranian Medical University, 70-204 Szczecin, Poland; asklepiada@wp.pl; 6 Multidiscyplinary Voivodship Hospital, 66-400 Gorzów Wlkp, Poland; 7 ID Clinic, 41-400 Mysłowice, Poland; e.janczewska@poczta.fm; 8 Department of Infectious Diseases and Hepatology, Faculty of Medicine, Collegium Medicum in Bydgoszcz, Nicolaus Copernicus University, 85-030 Bydgoszcz, Poland; d.dybowska@wsoz.pl; 9 Department of Infectious Diseases and Hepatology, Medical University of Bialystok, 15-540 Białystok, Poland; anna.parfieniuk@gmail.com (A.P.-K.); robert.flisiak1@gmail.com (R.F.); 10 Department of Infectious Diseases, Medical University of Lublin, 20-059 Lublin, Poland; magdalena.tudrujek@gmail.com; 11 Biegański Regional Specjalist Hospital, 91-347 Łódź, Poland; jolanta.e.bialkowska@gmail.com; 12 Infectious Diseases and Hepatology Clinic, Medical University of Wroclaw, 50-367 Wrocław, Poland; justyna@janocha-litwin.pl

**Keywords:** chronic hepatitis C, hepatitis C virus, liver transplantation, direct-acting antivirals

## Abstract

**Background/Objectives:** Direct-acting antivirals (DAAs) have revolutionized the management of chronic hepatitis C virus (HCV) infection. However, real-world data on the effectiveness and safety of DAA therapy in patients with history orthotopic liver transplantation (OLTx) remain limited. This study aimed to evaluate the clinical characteristics, effectiveness, and safety of DAA therapy in liver transplant recipients with chronic hepatitis C in a nationwide, real-world cohort. **Methods:** A retrospective analysis was performed of all consecutive adult patients who underwent OLTx before starting DAA therapy between July 2015 and December 2024 within the EpiTer-2 project, which included 20,586 patients treated because of chronic hepatitis C. **Results:** A total of 141 patients participated in the study, with most of them being men (66%) and aged 50 years or older. Most patients (80%) had comorbidities, and nearly a quarter of the population had cirrhosis of the transplanted liver at the start of antiviral therapy. The median time from OLTx to initiation of antiviral therapy was 24 months. Overall, SVR was achieved in 96.4% of patients in the intention-to-treat analysis and in 98.6% after excluding patients lost to follow-up. The treatment was well tolerated. Serious adverse events were reported in five patients. During DAA treatment and 12 weeks of follow-up after treatment, two deaths were reported. Subgroup analysis by time from OLTx to antiviral therapy (≤24 vs. >24 months) revealed no differences in effectiveness and safety despite some baseline clinical variations. **Conclusions:** DAA therapy in liver transplant recipients with chronic HCV infection is highly effective and well-tolerated.

## 1. Introduction

Hepatitis C virus (HCV) infection remains a major global health burden despite significant progress in treatment strategies. According to the World Health Organization’s estimates, more than 50 million people worldwide have chronic HCV infection, with over one million new infections occurring annually [1]. The chronic nature of the infection results in long-term hepatic inflammation and progressive liver injury, leading to cirrhosis and hepatocellular carcinoma (HCC) in a substantial proportion of patients [2,3]. Consequently, HCV continues to be one of the leading causes of liver-related morbidity [4,5]. Even in the era of modern antiviral therapy, chronic HCV infection remains one of the most frequent indications for orthotopic liver transplantation (OLTx) [6,7]. Historically, the management of HCV in liver transplant recipients has posed considerable clinical challenges. Reinfection of the liver graft with HCV was nearly universal in the pre-direct-acting antiviral (DAA) era, and recurrent infection was associated with an accelerated course of disease compared to immunocompetent non-transplant patients [8,9]. Rapid progression of fibrosis, deterioration of graft function, and higher rates of graft loss and patient mortality were commonly observed [10].

Interferon-based antiviral therapies, previously the only available treatment option, were poorly tolerated in transplant recipients, largely due to hematological toxicity, risk of graft rejection, and numerous contraindications [11,12]. Their effectiveness was also unsatisfactory, with sustained virological response (SVR) rates not exceeding 30–40% in most series, leaving a large group of patients with no effective therapeutic option [12,13].

The introduction of DAAs marked a turning point in the treatment of HCV. These oral regimens directly inhibit specific viral proteins essential for viral replication and have demonstrated consistently high SVR rates, exceeding 95% in both clinical trials and real-world studies [14]. Treatment is typically short, well tolerated, and effective even in patients who were previously considered difficult to treat, including those with cirrhosis, renal impairment, or post-transplant immunosuppression [15]. The application of DAAs in liver transplant recipients has dramatically altered the natural course of HCV reinfection after OLTx, improving graft survival and overall patient prognosis to an unprecedented degree [16].

However, much of the current evidence supporting the use of DAAs in transplant populations originates from randomized clinical trials and large international or multicenter real-world cohorts [17,18,19,20,21]. While these studies provide valuable insights, they may not fully capture outcomes in routine clinical practice across different healthcare systems, particularly where access to drugs and structures of care vary. Real-world data are of particular importance in post-transplant patients, as they represent a complex group characterized by polypharmacy, multiple comorbidities, and ongoing immunosuppressive therapy that may interact with antiviral drugs. Although several cohorts from Western Europe and North America have been published, evidence from Central and Eastern Europe remains scarce. To date, no comprehensive analysis has been undertaken in Poland, despite the fact that all HCV therapies are uniformly reimbursed by the National Health Fund (NHF), creating unique conditions for population-wide evaluation.

In this context, the present study aimed to provide a detailed analysis of Polish liver transplant recipients treated with DAAs within the national EpiTer-2 database. Specifically, we sought to assess the clinical characteristics of this population, the effectiveness of antiviral therapy as measured by SVR, and the safety profile of DAAs in the real-world, post-transplant setting. This is the first large-scale national study addressing these issues in Poland and contributes to the broader European perspective on HCV management after liver transplantation.

## 2. Materials and Methods

The study population was selected from the non-interventional EpiTer-2 database, which includes 20,586 retrospectively collected records of patients treated for chronic hepatitis C between 1 July 2015 and 31 December 2024 at Polish hepatology centers. The analyzed population included all consecutive patients from this database who underwent liver transplantation before starting antiviral therapy.

No patients were excluded based on missing baseline data, reflecting the completeness and reliability of the dataset. Regarding coinfections, one patient had concurrent hepatitis B virus (HBV) infection; no patients had coinfection with HIV. No other exclusion criteria were applied, and all consecutive liver transplant recipients meeting the general inclusion criteria were included, which ensures that the study population represents real-world clinical practice. Patients with missing follow-up data were considered lost-to-follow-up (LTFU) for efficacy endpoints but were included in analyses of baseline characteristics.

Patients were treated for chronic hepatitis C under the reimbursed therapeutic program of the National Health Fund (NHF). The choice of the type of therapeutic regimen was made by the treating physician, guided by the characteristics of the product, the requirements of the drug program and the recommendations of international scientific societies and the Polish HCV Expert Group [22,23]. At the start of therapy, patients provided informed consent for treatment and processing of personal data in accordance with the requirements of the drug program and national regulations.

Patient data were retrospectively obtained from medical records and entered online into a web-based platform managed by Tiba sp. z o.o. Data collected at baseline of DAA treatment comprised demographic and clinical parameters, including comorbidities, HBV and human immunodeficiency virus (HIV) coinfections, and co-medications. Laboratory results, such as complete blood count, ALT activity, creatinine concentration, and parameters defining liver function, namely bilirubin, albumin, and international normalized ratio (INR), were also analyzed. Data characterizing HCV infection included virological test results, such as genotype and viral load of HCV ribonucleic acid (RNA) measured by real-time polymerase chain reaction with a lower detection limit of 15 IU/mL.

The initial evaluation provided data on the current severity of liver disease in terms of the presence of cirrhosis diagnosed by non-invasive methods, including transient elastography with FibroScan or shear wave elastography with Aixplorer. Elastographic findings in the form of liver stiffness expressed in kilopascals were presented in correlating degrees of fibrosis (F) on the Metavir scale according to European Association for the Study of the Liver (EASL) recommendations, and the F4 score was the basis for the diagnosis of cirrhosis [24]. Patients diagnosed with cirrhosis were assessed on the Child–Pugh (CP) scale, and data were collected on decompensated liver function in the form of ascites or encephalopathy, both in the past and at the start of DAA therapy. Data were reported on the reason for performing OLTx on patients, the time elapsed between surgery and DAA therapy, and the type of immunosuppression used.

Information on antiviral therapy included data on the history of previous treatment and the type of DAA regimen currently being used, as well as its effectiveness and safety. Patients received genotype-specific or pangenotypic DAA options. The measure of the effectiveness of the therapy was the achievement of an SVR, defined as a negative HCV RNA result in a test performed 12 weeks after the end of the therapy. Patients with detectable HCV RNA at this timepoint were virological non-responders, while those without SVR assessment were considered LTFU. Treatment safety was routinely assessed during therapy and 12 weeks after completion by collecting data on the course of treatment, incidence of adverse events (AEs), including serious adverse events (SAEs) and AEs of particular interest, such as ascites, encephalopathy, and gastrointestinal bleeding, and deaths. At the time of this analysis, in April 2025, retrospective data on patient survival and the incidence of HCC recurrence in patients transplanted for HCC were obtained.

Patients were additionally stratified into two subgroups according to the median time from OLTx to the initiation of antiviral therapy (≤24 months vs. >24 months). Baseline demographic and clinical characteristics, comorbidities, laboratory parameters, and treatment outcomes were compared between these groups.

### 2.1. Statistical Analysis

Categorical variables were summarized using frequency counts and percentages, with group comparisons assessed via Fisher’s exact test or the chi-square test, as appropriate. Due to the non-normal distribution of continuous variables, medians and interquartile ranges were reported, and the Mann–Whitney U test was used for between-group comparisons. The distribution of the data was evaluated with the Shapiro–Wilk test. All statistical procedures were carried out using Statistica version 13 (StatSoft, Tulsa, OK, USA) and GraphPad Prism version 5.1 (GraphPad Software, Inc., La Jolla, CA, USA).

### 2.2. Ethics

The study received ethical approval from the Bioethics Committee of Jan Kochanowski University in Kielce (Resolution No. 57/2024, dated 25 July 2024). Participation in the therapeutic program was voluntary, and all patients provided informed consent in line with the National Health Fund’s regulations.

## 3. Results

### 3.1. Study Population

**Figure 1:**
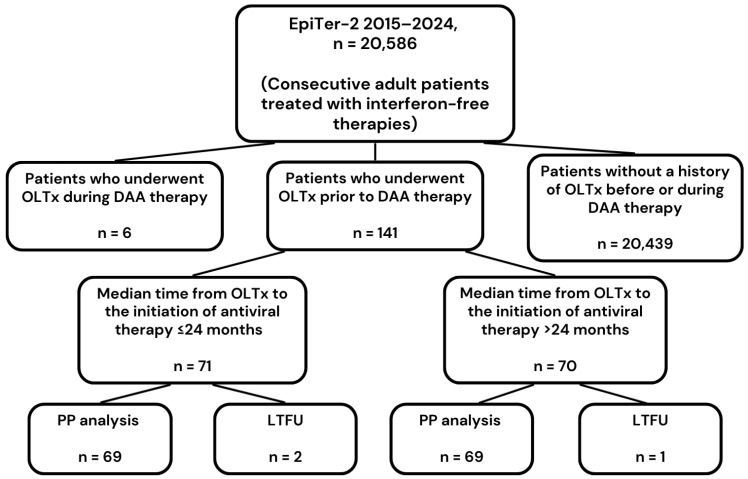
Study flowchart. Abbreviations: DAA, direct-acting antiviral; HCC, hepatocellular carcinoma; LTFU, loss to follow-up; OLTx, orthotopic liver transplantation; PP, per-protocol.

The study group included all consecutive patients with chronic HCV infection treated with direct-acting antiviral drugs who underwent liver transplantation before starting antiviral therapy. They were selected from a large national EpiTer-2 database of 20,586 adult patients treated with DAA from 1 July 2015 to 31 December 2024 at 22 Polish hepatology centers (Figure 1).

The analyzed population consisted of 141 patients, mostly men (66%), with three quarters aged 50 years and over (Table 1). One hundred and thirteen (80.1%) were burdened with comorbidities, the most common of which was hypertension, followed by diabetes and chronic renal disease, which in two patients was the reason for kidney transplantation. Disregarding immunosuppressive therapy after OLTx, nearly 70% of patients were taking other medications due to coexisting diseases. Only one patient was diagnosed with HBV infection, and no cases of HIV coinfection were identified.

Table 2 shows the laboratory variables of patients at the start of DAA therapy. Median values of parameters relating to liver function (albumin, bilirubin, INR) remained within normal limits.

The majority of patients had GT1b infection, and more than half of the population was treatment-experienced, mainly with interferon-based regimens (Table 3). During the current therapy, over 80% of patients received genotype-specific DAA options, the most commonly used of which were ombitasvir/paritaprevir/ritonavir ± dasabuvir ± ribavirin (OBV/PTV/r ± DSV ± RBV) and Sofosbuvir/ledipasvir ± ribavirin (SOF/LDV ± RBV), which were administered to 55 patients each.

Cirrhosis at the start of DAA therapy was diagnosed in 35 patients, corresponding to a quarter of the population, 10 of whom showed signs of decompensation in the form of ascites and/or hepatic encephalopathy (Table 4). A total of eleven patients with cirrhosis were rated as Grade B and one met the criteria for Grade C on the CP scale.

**Figure 2:**
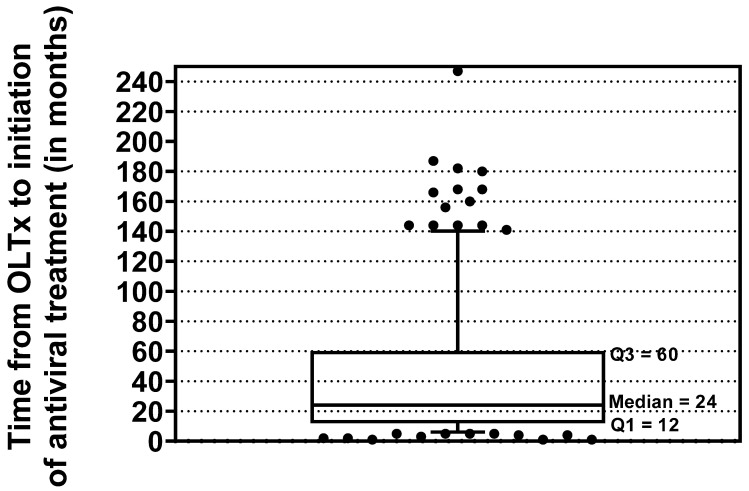
Time between OLTx and DAA treatment. Abbreviations: DAA, direct-acting antiviral; OLTx, orthotopic liver transplantation.

The median time (IQR) [min-max] from OLTx to initiation of antiviral treatment was 24 (12–60) [1–247] months (Figure 2). Most patients (*n* = 122) received tacrolimus as immunosuppressive therapy; only 19 individuals (13.4%) were on cyclosporin. Selection of DAA regimen was made each time after analyzing potential drug–drug interactions according to the recommendations using the Liverpool Hep Drug Interaction Checker (EASL).

### 3.2. Treatment Safety

The majority of patients completed the treatment course as planned (Table 5). About 57% of the patients experienced at least one AE, the most commonly reported of which was fatigue. Most AEs were mild in intensity; SAEs (significant increase in ALT activity, HCC dissemination, liver cirrhosis decompensation in two patients, increase in alpha-fetoprotein concentration) were reported in five patients. In two patients, SAEs were the reason for discontinuation of therapy; in one patient, this was due to HCC dissemination (after 12 weeks of a 24-week DAA regimen), and in another due to decompensation of liver function, but this patient finally responded to treatment. AEs of special interest, such as ascites, encephalopathy, and gastrointestinal bleeding, were noted in five, one, and one patient, respectively. Two patients died, both having undergone transplantation for HCC prior to DAA treatment; in one, who died during the follow-up period after discontinued treatment, the cause of death was HCC dissemination, while the death of the other (during treatment) was due to late vascular complications after OLTx.

### 3.3. Treatment Effectiveness

**Figure 3:**
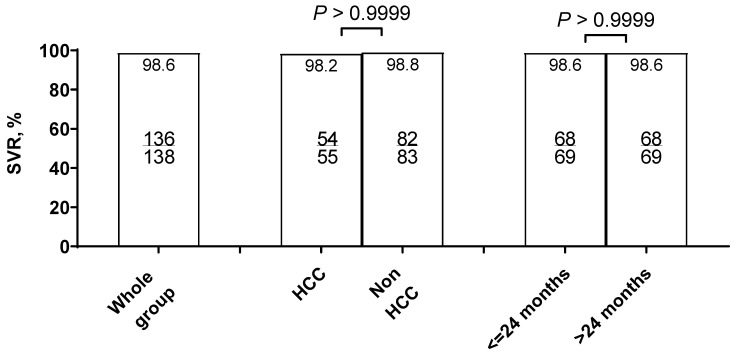
Effectiveness of DAA therapy overall and with consideration of the reason for liver transplantation and time between OLTx and DAA treatment (<=24 months and >24 months). Abbreviations: DAA, direct-acting antiviral; HCC, hepatocellular carcinoma; SVR, sustained virological response.

In two patients who died during the treatment and follow-up period, HCV RNA was not determined 12 weeks after the end of therapy; in addition, one more patient did not attend a scheduled visit at this follow-up point. Thus, the number of patients that were LTFU was three. Overall, the SVR rate in the analyzed population calculated according to intention-to-treat (ITT) analysis was 96.5% (137/142), and it was 98.6% (137/139) after excluding those LTFU (Figure 3). In the ITT analysis, there was no statistically significant difference in the SVR rate between patients with HCC and those without HCC (93.2% vs. 98.8%). Likewise, no statistically significant difference was observed in patients treated within ≤24 months after HCC therapy compared with those treated >24 months (95.8% vs. 97.1%). In the per-protocol (PP) analysis, no statistically significant differences between these groups were observed either.

Both virologic non-responders had GT1b infection and had been diagnosed with cirrhosis at baseline of DAA therapy. One was a treatment-naïve 47-year-old male transplanted for decompensated cirrhosis one year before 12-week therapy with OBV/PTV/r + DSV (2016), while the other was a 59-year-old female transplanted ten years before 12-week SOF + SMV treatment (2016) for HCC and had previously been treated with PegIFN + RBV.

### 3.4. Comparison of Subpopulations Depending on the Time from Liver Transplantation to the Start of Antiviral Therapy

The study population was stratified according to the median time from OLTx to the initiation of antiviral therapy (≤24 months vs. >24 months). These two groups did not differ with respect to baseline demographic characteristics. However, diabetes mellitus was observed significantly more frequently in the ≤24-month group. No significant differences were found in baseline laboratory parameters between the groups. Patients in the ≤24-month group were more often treatment-naïve, and HCC as an indication for OLTx was also more common in this subgroup. Importantly, there were no differences between the groups in terms of treatment efficacy or safety profile.

## 4. Discussion

In this multicenter, real-world study, we analyzed the clinical characteristics, treatment effectiveness, and safety of direct-acting antivirals in Polish liver transplant recipients with chronic HCV infection. To our knowledge, this is the first large-scale analysis of this patient group in Poland, providing valuable insights into real-world post-transplant management of HCV infection. In this context, real-world data from multicenter cohorts provide important insights into the feasibility, effectiveness, and tolerability of DAA therapy in routine medical practice, complementing evidence from controlled clinical trials.

Our analysis included patients selected from a large national cohort who had been treated with DAA therapy over a ten-year period using different DAA regimens depending on their current availability and the recommendations in force at the time. Their common feature was liver transplantation prior to DAA therapy, and out of a population of over 20,000, 141 patients met this criterion. Most of them underwent transplantation due to end-stage liver disease without cancer, and about 40% already had HCC; these are the two conditions that are the reason for liver transplantation in HCV infection [25]. In the analyzed population, regardless of the reason for liver transplantation, men predominated. This is due to the well-documented influence of male sex on the progression of liver disease in HCV infection and is consistent with reports by other authors of faster progression of liver disease in men and their higher share in the population of transplant patients [26,27].

The time interval between liver transplantation and the start of DAA therapy in our population was very wide, with a median (IQR) of 25 (12–60) months. In 34 patients (nearly a quarter of the group), reinfection with HCV during this period led to the development of cirrhosis, with 10 of them developing organ failure at the start of DAA therapy. This is consistent with available data from the literature, according to which approximately 30% of transplant recipients with HCV reinfection develop cirrhosis within 5 years of surgery [8,28].

The vast majority of patients in the analyzed population had GT1b infection, which is the most common HCV genotype in our country [29]. This phenomenon explains the significant advantage of genotype-specific regimens used in patients in our study. Slightly more than half of the patients had a history of unsuccessful antiviral therapy, mostly based on IFN. This relatively low percentage is due to the fact that access to antiviral treatment, both for patients with end-stage liver disease and patients after liver transplantation, was very limited during the IFN era due to safety concerns [14,29,30].

The results of treatment for HCV reinfection after liver transplantation were also poor, which consequently had a negative impact on the survival of transplant patients [8,31]. The introduction of DAAs has radically changed this landscape, offering high cure rates with excellent safety profiles. A retrospective cohort study of 226 liver transplant recipients conducted between 2007 and 2018 highlights this paradigm shift [32]. Patients transplanted after 2014, when DAAs became widely available, achieved substantially higher SVR rates compared with those transplanted in the pre-DAA era (86.7% vs. 15.4% at 2 years). Importantly, fibrosis progression, which was pronounced in the pre-DAA cohort prior to SVR, was essentially halted in the DAA era, underscoring the clinical impact of viral eradication on graft health and long-term liver function.

In our cohort of liver transplant recipients, DAA therapy achieved high SVR rates—96.5% in the intention-to-treat analysis and 98.6% after excluding patients lost to follow-up. These findings are consistent with previous studies, collectively demonstrating the reliable efficacy of DAA therapy in post-transplant settings. For example, the HCV-TARGET study—a large multicenter cohort of 347 liver transplant recipients treated with DAA—reported an SVR rate of 96.3% [33]. Similarly, a meta-analysis of 16 studies including 885 liver transplant recipients with genotype 1 found a pooled SVR12 rate of 93%, with regimens such as sofosbuvir/ledipasvir (SOF/LDV) showing the highest efficacy and tolerability [34]. However, not all real-world data are uniformly optimistic. A Latin American cohort reported a markedly increased risk of treatment failure among liver transplant recipients, who had nearly a fourfold higher likelihood of failing therapy compared with non-transplanted patients (OR 3.75, *p* = 0.01) [35]. This highlights the importance of individualized management, careful monitoring for drug–drug interactions, and early intervention in patients with advanced disease or other risk factors for suboptimal response. Despite the overall high efficacy of DAAs in liver transplant recipients, several factors have been associated with an increased risk of virologic failure. Advanced liver disease, particularly cirrhosis and decompensated liver function, remains a major predictor of suboptimal response, as these patients often have impaired hepatic metabolism and altered drug pharmacokinetics [35,36]. The presence of HCC has also been linked to lower SVR rates in some real-world cohorts, potentially due to underlying tumor-related immune dysregulation and hepatic impairment [35,37]. In line with these observations, the current analysis of Polish liver transplant recipients treated with DAAs confirmed that non-response was observed in patients with more advanced liver disease.

In our cohort, the majority of liver transplant recipients completed the planned DAA therapy, with approximately 57% experiencing at least one AE. Fatigue was the most commonly reported AE, which aligns with findings from other real-world studies of post-transplant DAA therapy. For example, the HCV-TARGET study reported fatigue in about 30% of patients, indicating that mild, non-serious AEs are common but generally well-tolerated in this population [33]. SAEs were relatively uncommon in our cohort, occurring in five patients. These included significant elevation activity of ALT, decompensation of liver cirrhosis, HCC dissemination, and increased alpha-fetoprotein concentration. Only two SAEs led to therapy discontinuation, highlighting the overall favorable safety profile of DAAs even in high-risk post-transplant patients. Importantly, two deaths were observed in patients with prior HCC; one was related to HCC dissemination after treatment discontinuation, and the other resulted from late post-transplant vascular complications. The HCV-TARGET study reported a slightly higher incidence of SAEs (11.9%) among post-liver transplant patients treated with IFN-free DAA regimens compared with our cohort [19]. A critical aspect of post-transplant DAA therapy is the potential for drug–drug interactions, particularly with immunosuppressive agents such as tacrolimus and cyclosporine. In our cohort, most patients received tacrolimus, and no clinically significant interactions leading to severe toxicity were observed, reflecting careful regimen selection and monitoring. This finding is consistent with prior studies emphasizing the need for individualized dosing and regular monitoring of immunosuppressant levels to minimize toxicity while maintaining antiviral efficacy [38,39]. Furthermore, the high prevalence of comorbidities and concomitant medications in our cohort highlights the importance of structured approaches to drug interaction management in the post-transplant setting, particularly in patients with cardiovascular or renal disease. Overall, the safety profile observed in our study confirms that DAAs are generally well-tolerated in liver transplant recipients, with most AEs being mild and manageable. However, special attention should be paid to patients with a history of HCC, advanced liver disease, or complex immunosuppressive regimens, as they remain at higher risk for serious complications.

In our cohort, stratification according to the time from OLTx to antiviral therapy initiation revealed only limited differences between groups. The higher proportion of treatment-naïve individuals and HCC as an indication for OLTx in this subgroup is consistent with the clinical context of early post-transplant management. Importantly, despite these baseline differences, no disparities were observed in treatment effectiveness and safety, underscoring the robustness of antiviral therapy across different post-transplant timelines. According to the EASL guidelines, antiviral therapy after liver transplantation should be initiated as early as possible once the patient’s condition is stabilized, typically within the first three months post-transplant [22]. The guidelines also emphasize that delaying treatment may be associated with lower SVR12 rates, particularly in patients with more advanced graft liver disease, which further supports the relevance of evaluating treatment outcomes in relation to the timing of DAA initiation.

This study has several important strengths. It represents the first large-scale analysis of Polish liver transplant recipients treated with DAAs, providing comprehensive real-world data from multiple hepatology centers across the country. An additional strength is the context of a unified national reimbursement system. Finally, the study benefited from a low rate of loss to follow-up, enhancing the reliability of the reported sustained virological response and safety outcomes. Despite these strengths, several limitations should be acknowledged. First, this study’s retrospective design inherently carries the risk of selection and reporting biases. Data were obtained from medical records, which may be incomplete or inconsistently documented across centers, potentially affecting the accuracy of baseline characteristics, comorbidities, and adverse event reporting. The heterogeneity of DAA regimens and treatment durations, while reflecting real-world clinical practice, prevents controlled evaluation of regimen-specific effects. Due to the observational design and limited sample size, group comparisons were presented without adjustment for potential confounders. As a result, baseline differences between the groups, such as the prevalence of diabetes, may influence the observed outcomes.

## 5. Conclusions

In this real-world national cohort, we documented a high effectiveness, exceeding 96%, and an acceptable safety profile of DAA therapy in liver transplant recipients with chronic HCV infection. No differences in treatment outcomes were observed depending on the time elapsed between OLTx and the start of DAA therapy. The high rate of HCV eradication after liver transplantation confirms the clinical benefits of antiviral therapy and highlights the importance of timely access to treatment in this population.

## Figures and Tables

**Table 1:** Characteristics of the study population.

Parameter	All, *n* = 141	≤24 Months, *n* = 71	>24 Months, *n* = 70	*p*
Age, median (IQR)	58.0 (49–63)	56 (49–61)	59 (52–63)	0.0842
Age ≥ 50, n (%)	105 (74.5)	49 (69)	56 (80)	0.1347
Gender, men/women, n (%)	93 (66)/ 48 (34)	47 (66.2)/ 24 (33.8)	46 (65.7)/ 24 (34.3)	0.9517
BMI, median (IQR)	25.9 (23.9–28.7)	25.8 (23.9–28.8)	26.5 (23.5–28.7)	0.9291
Comorbidities, n (%)				
Any comorbidity	113 (80.1)	56 (78.9)	57 (81.4)	0.7037
Diabetes	58 (41.1)	35 (49.3)	23 (32.9)	0.0473
Obesity	22 (15.6)	10 (14.1)	12 (17.1)	0.6142
Arterial hypertension	66 (46.8)	33 (46.5)	33 (47.1)	0.937
Autoimmune disease	1 (0.7)	0	1 (1.4)	0.4964
Non-HCC tumors	3 (2.1)	0	3 (4.3)	0.1197
Renal disease/kidney transplantation	48 (34)/2 (1.4)	19 (26.8)	29 (41.4)	0.0661
Concomitant medications (apart from immunosuppressants), n (%)	98 (69.5)	48 (67.6)	50 (71.4)	0.622
HBV coinfection (HBsAg+), n (%)	1 (0.7)	1	0	0.4091
HIV coinfection, n (%)	0	0	0	NA

Abbreviations: BMI, body mass index; HBsAg, hepatitis B surface antigen; HBV, hepatitis B virus; HCC, hepatocellular carcinoma; HIV, human immunodeficiency virus; IQR, interquartile range; NA, not applicable.

**Table 2:** Baseline laboratory parameters.

Parameter	All, *n* = 141	≤24 Months, *n* = 71	>24 Months, *n* = 70	*p*
ALT (IU/L), median (IQR)	60 (40.5–106)	62 (43–122)	56 (35–94)	0.1504
Albumin (g/dL), median (IQR)	4.1 (3.8–4.5)	4.1 (3.8–4.5)	4.1 (3.8–4.4)	0.9881
Bilirubin (mg/dL), median (IQR)	1 (0.7–1.3)	1 (0.8–1.4)	0.9 (0.6–1.3)	0.1575
Hemoglobin (g/dL), median (IQR)	14 (12.8–15.2)	14.3 (12.8–15.2)	14 (12.8–15.3)	0.8995
Platelets (×1000/µL), median (IQR)	145.5 (103–170)	131 (93–168)	148 (114.5–178)	0.2535
Creatinine (mg/dL), median (IQR)	1.0 (0.8–1.2)	1 (0.8–1.1)	1 (0.8–1.2)	0.1342
INR, median (IQR)	1 (1–1.1)	1 (1–1.1)	1 (1–1.1)	0.7306
HCV RNA, ×10^6^ IU/mL, median (IQR)	1.6 (0.6–3.6)	1.6 (0.8–3.9)	1.5 (0.5–3.4)	0.4409

Abbreviations: ALT, alanine aminotransferase; HCV, hepatitis C virus; INR, international normalized ratio; IU, international units; RNA, ribonucleic acid; µL, microliter; IQR, interquartile range.

**Table 3:** Characteristics of HCV infection and antiviral therapy.

Parameter	All, *n* = 141	≤24 Months, *n* = 71	>24 Months, *n* = 70	*p*
HCV genotype, n (%)				0.1021
1a	1 (0.7)	0	1 (1.4)	
1b	123 (87.2)	58 (81.7)	65 (92.9)	
1	3 (2.2)	1 (1.4)	2 (2.9)	
3	11 (7.8)	9 (12.7)	2 (2.9)	
4	2 (1.4)	2 (2.8)	2 (2.9)	
Unknown	1 (0.7)	1 (1.4)	0	
History of antiviral therapy, n (%)				
Treatment-naive	67 (47.5)	40 (56.3)	27 (38.6)	0.0347
Treatment-experienced, non-responder to IFN-based regimens	66 (46.8)	25 (35.2)	42 (60)	0.0032
Treatment-experienced, non-responder to IFN-free regimens	8 (5.7)	6 (8.5)	1 (1.4)	0.1157
Current treatment regimen, n (%)				
Genotype-specific treatment regimens	116 (82.3)	55 (77.5)	61 (87.1)	0.1325
SOF/LDV ± RBV	55 (39)	25 (35.2)	30 (42.9)	0.352
OBV/PTV/r ± DSV ± RBV	55 (39)	28 (39.4)	27 (38.6)	0.9161
GZR/EBR ± RBV	3 (2.1)	1 (1.4)	2 (2.9)	0.6196
SOF + SMV ± RBV	3 (2.1)	1 (1.4)	2 (2.9)	0.6196
Pangenotypic regimens	25 (17.7)	16 (22.5)	9 (12.9)	0.1325
SOF + RBV	6 (4.3)	5 (7)	1 (1.4)	0.2087
SOF + DCV ± RBV	5 (3.5)	2 (2.8)	3 (4.3)	0.6807
SOF/VEL ± RBV	10 (7.1)	5 (7)	5 (7.1)	>0.9999
SOF/VEL/VOX	4 (2.9)	4 (5.6)	0	0.1197

Abbreviations: DAA, direct-acting antiviral; DCV, Daclatasvir; EBV, Elbasvir; GZR, Grazoprevir; IFN, Interferon; LDV, Ledipasvir; OBV, Ombitasvir; PTV, Paritaprevir; RBV, Ribavirin; SMV, Simeprevir; SOF, Sofosbuvir; VEL, Velpatasvir; VOX, Voxilaprevir; HCV, hepatitis C virus.

**Table 4:** Characteristics of liver disease severity and liver transplantation.

Parameter	All, *n* = 141	≤24 Months, *n* = 71	>24 Months, *n* = 70	*p*
Liver fibrosis at the start of therapy, n (%)				0.6313
F0	7 (5)	4 (5.6)	3 (4.3)	
F1	37 (26.2)	22 (31)	15 (21.4)	
F2	36 (25.5)	8 (11.3)	20 (28.6)	
F3	18 (12.8)	15 (21.1)	10 (14.3)	
F4	34 (24.1)	16 (22.5)	19 (27.1)	
No data	9 (6.4)	6 (8.5)	3 (4.3)	
Liver decompensation at baseline, n (%) Ascites Encephalopathy	10 (7.1) 9 (6.4) 3 (2.1)	5 (7) 4 (5.6) 1 (1.4)	5 (7.1) 5 (7.1) 2 (2.9)	>0.9999 0.743 0.6169
Child–Pugh, in relation to F4, % (n)				
B, % (n)	11 (32.4)	6 (37.5)	5 (26.3)	0.7159
C, % (n)	1 (2.9)	0	1 (5.3)	>0.9999
Indication for OLTx before DAA therapy, n (%)				
Cirrhosis	81 (57.5)	31 (43.7)	50 (70.4)	0.0009
HCC	58 (41.1)	38 (53.5)	20 (28.6)	0.0026

Abbreviations: F, fibrosis stage (Metavir score); HCC, hepatocellular carcinoma; OLTx, orthotopic liver transplantation; DAA, direct-acting antiviral.

**Table 5:** Safety of DAA therapy in patients after liver transplantation.

Parameter	All, *n* = 141	≤24 Months, *n* = 71	>24 Months, *n* = 70	*p*
Treatment course, n (%)				0.2088
According to schedule	123 (87.2)	60 (84.5)	63 (90)	
Therapy modification (RBV dose)	15 (10.7)	8 (11.3)	7 (10)	
Therapy discontinuation	3 (2.1)	3 (4.2)	0	
Serious AEs, n (%)	5 (3.5)	4 (5.6)	1 (1.4)	0.366
AEs leading to treatment discontinuation, n (%)	2 (1.4)	2 (2.8)	0	0.4964
Patients with at least one AE, n (%)	81 (57.4)	42 (59.2)	39 (55.7)	0.6795
Weakness/fatigue	45 (31.9)	21 (29.6)	24 (34.3)	0.5487
Anemia	30 (21.3)	14 (19.7)	16 (22.9)	0.6489
Headache	12 (8.5)	7 (9.9)	5 (7.1)	0.7643
Itchy skin	3 (2.1)	1 (1.4)	2 (2.9)	0.6196
AEs of particular interest, n (%)				
Ascites	5 (3.5)	2 (2.8)	3 (4.3)	0.6807
Hepatic encephalopathy	1 (0.7)	0	1 (1.4)	0.4964
Gastrointestinal bleeding	1 (0.7)	0	1 (1.4)	0.4964
Death, n (%)	2 (1.4)	2 (2.8)	0	0.4964

Abbreviations: AE, adverse event; RBV, Ribavirin.

## Data Availability

The data are available through the SARSTer database, https://www.epiter-2.pl/ (accessed on 10 August 2025), after obtaining the coordinator’s approval.

## Abbreviations

The following abbreviations are used in this manuscript:

AE	Adverse event
CP	Child–Pugh
DAA	Direct-acting antiviral
EASL	European Association for the Study of the Liver
F	Metavir fibrosis score
HBV	Hepatitis B virus
HCC	Hepatocellular carcinoma
HCV	Hepatitis C virus
HIV	Human immunodeficiency virus
INR	International normalized ratio
LTFU	Lost-to-follow-up
NHF	National Health Fund
OBV/PTV/r ± DSV ± RBV	Ombitasvir/paritaprevir/ritonavir ± dasabuvir ± ribavirin
OLTx	Orthotopic liver transplantation
SOF/LDV	Sofosbuvir/ledipasvir
SOF/LDV ± RBV	Sofosbuvir/ledipasvir ± ribavirin
SVR	Sustained virological response
